# Association between pulmonary function and renal function: findings from China and Australia

**DOI:** 10.1186/s12882-017-0565-y

**Published:** 2017-05-01

**Authors:** Dahai Yu, Tao Chen, Yamei Cai, Zhanzheng Zhao, David Simmons

**Affiliations:** 1 Department of Nephrology, The First Affiliated Hospital, Zhengzhou University, Zhengzhou, 450052 China; 2 Arthritis Research UK Primary Care Centre, Research Institute for Primary Care and Health Sciences, Keele University, Keele, ST5 5BG UK; 3 Division of Health and Social Care, King College London, London, SE1 3QD UK; 4 Western Sydney University, Locked Bag 1797, Campbelltown, Sydney, NSW 2751 Australia

**Keywords:** Forced expiratory volume in one second (FEV1), Forced vital capacity (FVC), Lung capacity, Renal function, Estimated glomerular filtration rate, The predicted percentage value of forced expiratory volume in 1 s (PFEV1), The predicted percentage value of forced vital capacity (PFVC)

## Abstract

**Background:**

The relationship between obstructive lung function and impaired renal function is unclear. This study investigated the dose-response relationship between obstructive lung function and impaired renal function.

**Methods:**

Two independent cross-sectional studies with representative sampling were applied. 1454 adults from rural Victoria, Australia (1298 with normal renal function, 156 with impaired renal function) and 5824 adults from Nanjing, China (4313 with normal renal function, 1511 with impaired renal function). Pulmonary function measurements included forced expiratory volume in one second (FEV1) and forced vital capacity (FVC). Estimated glomerular filtration rate (eGFR), and impaired renal function marked by eGFR <60 mL/min/1.73m^2^ were used as outcome.

**Results:**

eGFR increased linearly with FEV1 in Chinese participants and with FVC in Australians. A non-linear relationship with peaked eGFR was found for FEV1 at 2.65 L among Australians and for FVC at 2.78 L among Chinese participants, respectively. A non-linear relationship with peaked eGFR was found for the predicted percentage value of forced expiratory volume in 1 s (PFEV1) at 81–82% and for the predicted percentage value of forced vital capacity (PFVC) at 83–84% among both Chinese and Australian participants, respectively.

The non-linear dose-response relationships between lung capacity measurements (both for FEV1 and FVC) and risk of impaired renal function were consistently identified in both Chinese and Australian participants. An increased risk of impaired renal function was found below 3.05 L both for FEV1 and FVC, respectively. The non-linear relationship between PFEV and PVC and the risk of impaired renal function were consistently identified in both Chinese and Australian participants. An increased risk of impaired renal function was found below 76–77% for PFEV1 and 79–80% for PFVC, respectively.

**Conclusions:**

In both Australian and Chinese populations, the risk of impaired renal function increased both with FEV1 and FVC below 3.05 L, with PFEV1 below 76–77% or with PFVC below 79–80%, respectively. Obstructive lung function was associated with increased risk of reduced renal function. The screen for impaired renal function in patients with obstructive lung disease might be useful to ensure there was no impaired renal function before the commencement of potentially nephrotoxic medication where indicated (eg diuretics).

**Electronic supplementary material:**

The online version of this article (doi:10.1186/s12882-017-0565-y) contains supplementary material, which is available to authorized users.

## Background

The kidney works with the heart, liver, lungs and other organs to maintain whole-body homeostasis [1]. Studies have established associations between the kidney and other organs, especially the heart through organ-to-organ networks, including various humoral factors, and neuronal network systems [2]. Few studies have investigated the association between the kidney and the lungs.

The National Health and Nutrition Examination Survey (NHANES) 2007–2012 investigated obstructive and restricted lung function measures and chronic kidney disease among people aged 40 to 79 years and revealed that estimated glomerular filtration rate (eGFR) < 60 mL/min/1.73 m^2^ was associated with higher odds of obstructive lung function [3]. However so far few studies has addressed the dose-response relationship between restrictive lung function and kidney function.

In this study, we aimed to explore the quantitative association between lung function measures, forced expiratory volume in one second (FEV1) and forced vital capacity (FVC) and kidney function measured by eGFR. Furthermore, our study aimed to evaluate the dose-response relationship between lung function measures and risk of restricted renal function in a representative Chinese population and then replicate this in an Australian population.

## Methods

The Chinese participants were from the Nanjing Community Cardiovascular Risk Survey and the Australian participants were from the Crossroads study. The sampling methods and the recruitment of participants of two studies were described in previous publications [4, 5–8].

Briefly, in both surveys, information on age, gender, smoking status, comorbidities such as asthma, chronic pulmonary disease and diagnosed diabetes were obtained through questionnaires by face-to-face interviews managed by trained research staff. Systolic and diastolic blood pressure, waist circumference, height and weight were measured 3 times and the arithmetic mean of the 2 closest measurements was recorded as the final measurement. Lung capacity including both forced expiratory volume 1 s and forced vital capacity were measured in accordance with American Thoracic Society guidelines [8] in both surveys. The detailed method of lung capacity measurement was described in the previous publication [4]. We also estimated the predicted forced vital capacity and forced expiratory volume in one second using the method developed by Carpo [9]. We used the predicted percentage value of forced expiratory volume in 1 s (PFEV1) and the predicted percentage value of forced vital capacity (PFVC) as another two measurements of lung capacity in this study.

Blood sampling methodology [9], fasting glucose and lipid profile measurement methodology [4], the definitions of type 2 diabetes [6], metabolic syndrome (MS) [10], and hypertension [11, 12] were reported previously. Patients with both type 2 diabetes and MS were classified ashaving type 2 diabetes only.

The eGFR was calculated from serum creatinine using the Chronic Kidney Disease Epidemiology Collaboration (CKD-EPI) eq. [13]. Reduced renal function was defined as eGFR <60 ml/min/1.73m^2^ [3]. Due to non-standardised creatinine measurement, (Isotope Dilution Mass Spectrometry (IDMS) standardised creatinine assay) adjusted creatinine was applied in the eGFR estimation in the Crossroads study [14].

We applied Kruskal–Wallis test and chi-squared test to compare continuous variables and categorical variables, respectively. The dose-response relationships between lung capacity measures (FEV1 and FVC) and eGFR were analysed by multiple linear regression model. We applied unconditional Logistic regression model with linear rem of lung capacity measurements, a natural cubic spline model with four equally spaced knots determined from the levels of lung capacity measurements, and a quadratic spline model [15, 16] to investigate the dose-response association between lung capacity measurements and the odds ratios of decreased renal function. The minimum Akaike information criterion (AIC) [17] was estimated in each model to detect the best-fit model, which chose that natural cubic spline model as the best-fit model.

We applied break-point test by including the piecewise term into the final model to hunt the potential threshold within the 5th percentile (P5) to the 95th percentile (P95) of lung capacity measures [18]. The final threshold was defined the threshold with a significant break in the regression coefficients and achieving the minimum AIC [12]. 1000 bootstrapping was applied to estimate the 95% confidence interval of the final threshold [15].

We applied several sensitivity analyses. First, we tested final model (natural cubic spline model) for the overall dataset and examined other potential knots within the range (minimum to maximum) measure of lung capacity [12]. We applied the final model in the data rich range (5th percentile to the 95th percentile) of lung capacity as the second sensitivity analysis [12]. We also applied the linear test in the final model to investigate the linearity of the dose-response relationship [19]. Finally, the dose-response association between obstructive lung function (FEV1/FVC < 0.70) [20] and reduced renal function was analysed in the general population, people with normal glucose metabolism status, and those with MS or type 2 diabetes, respectively.

Due to the cluster sampling design, the sampling weights were adjusted in all analyses of Nanjing data with allocation of ‘svy’ synax in Stata.

All analyses were processed by STATA 13 with statistical significance defined by two-tailed *P* < 0.05.

The Crossroads study was approved by the Goulburn Valley Health Ethics Committee (GCH-3/99). The Nanjing Community Cardiovascular Risk Survey was approved by the Institutional Review Board of Jiangsu Province Hospital on Integration of Chinese and Western Medicine (11–006). Signed, informed consent was obtained from all participants in both surveys.

## Results

People with reduced renal function (eGFR <60 ml/min/1.73m^2^) were more likely to be older, be a current smoker and have higher waist circumference, systolic blood pressure, triglyceride with lower lung capacity both in Chinese and Australian populations (Table 1).

**Table 1:** Characteristics of participants in all and by renal function status

	All	eGFR ≥ 60 mL/min/1.73 m^2^	eGFR < 60 mL/min/1.73 m^2^	*P*-value
Nanjing survey				
Participants, n	5824	4313	1511	
Age, years	52.0 (43.0 to 59.0)	51.0 (43.0 to 58.0)	55.0 (48.0 to 62.0)	0.0001
Women, %	56.3%	47.8%	67.5%	<0.0001
Current smoking, %	28.0%	18.3%	66.4%	<0.0001
Asthma/COPD, %	1.1%	1.1%	1.1%	0.9720
Body mass index, kg/m^2^	23.6 (21.4 to 26.1)	23.6 (21.4 to 26.1)	23.6 (21.4 to 26.1)	0.5862
Waist circumference, cM	80.0 (73.3 to 87.0)	79.3 (73.0 to 86.2)	82.5 (75.0 to 89.5)	0.0001
Systolic blood pressure, mmHg	128.0 (116.3 to 142.5)	126.5 (115.0 to 141.0)	131.0 (120.0 to 145.5)	0.0001
Diastolic blood pressure, mmHg	80.5 (73.5 to 88.5)	80.0 (72.5 to 88.0)	82.0 (75.0 to 90.5)	0.0001
Fasting glucose, mmol/L	5.4 (4.9 to 5.9)	5.4 (4.9 to 5.9)	5.7 (5.0 to 7.1)	0.0698
Triglyceride, mmol/L	1.2 (0.8 to 1.7)	1.2 (0.8 to 1.7)	1.2 (0.8 to 1.9)	0.0001
Total cholesterol, mmol/L	4.4 (3.9 to 4.9)	4.4 (3.9 to 5.0)	4.4 (3.9 to 4.9)	0.0783
High density lipoprotein cholesterol, mmol/L	1.3 (1.1 to 1.5)	1.3 (1.1 to 1.6)	1.2 (1.1 to1.5)	0.0001
Low density lipoprotein cholesterol, mmol/L	2.4 (2.0 to 2.9)	2.4 (2.0 to 2.8)	2.4 (2.1 to 2.9)	0.3603
Forced expiratory volume in 1 s, L	2.9 (2.6 to 3.4)	3.3 (2.9 to 3.7)	2.8 (2.5 to 3.2)	0.0001
Forced vital capacity, L	2.8 (2.4 to 3.4)	3.3 (2.8 to 3.8)	2.7 (2.3 to 3.2)	0.0001
Creatinine, mg/dl	1.0 (0.9 to 1.1)	0.9 (0.8 to 1.0)	1.2 (1.1 to 1.3)	0.0001
Crossroads				
Participants, n	1454	1298	156	
Age, years	52.0 (40.0 to 65.0)	50.0 (39.0 to 61.0)	76.0 (70.8 to 79.3)	0.0001
Women, %	56.0%	53.7%	56.3%	0.5660
Current smoking, %	18.9%	6.7%	20.2%	<0.0001
Asthma/COPD, %	14.3%	13.4%	14.6%	0.7240
Body mass index, kg/m^2^	27.0 (24.3 to 30.7)	27.0 (24.3 to 30.6)	27.4 (24.5 to 31.6)	0.3803
Waist circumference, cM	94.5 (84.5 to 104.3)	94.2 (84.3 to 103.9)	97.6 (86.1 to 109.3)	0.0075
Systolic blood pressure, mmHg	130.0 (114.0 to145.0)	128.5 (113.0 to 142.5)	147.0 (130.9 to 162.6)	0.0001
Diastolic blood pressure, mmHg	72.0 (65.0 to 79.0)	71.0 (63.0 to 76.0)	72.3 (65.5 to 80.5)	0.4244
Fasting glucose, mmol/L	5.1 (4.8 to 5.5)	5.0 (4.7 to 5.4)	5.3 (4.9 to 5.9)	0.0001
Triglyceride, mmol/L	1.2 (0.9 to 1.7)	1.2 (0.8 to 1.7)	1.4 (1.1 to 1.9)	0.0001
Total cholesterol, mmol/L	5.2 (4.6 to 5.9)	5.1 (4.4 to 5.9)	5.2 (4.6 to 5.9)	0.3340
High density lipoprotein cholesterol, mmol/L	1.4 (1.1 to 1.6)	1.4 (1.2 to 1.6)	1.3 (1.1 to 1.7)	0.9405
Low density lipoprotein cholesterol, mmol/L	3.1 (2.6 to 3.7)	3.0 (2.3 to 3.7)	3.2 (2.6 to 3.7)	0.1003
Forced expiratory volume in 1 s, L	2.8 (2.3 to 3.4)	2.9 (2.4 to 3.5)	1.9 (1.5 to 2.8)	0.0001
Forced vital capacity, L	3.5 (2.9 to 4.3)	3.6 (3.0 to 4.4)	2.5 (2.1 to 3.3)	0.0001
Creatinine, mg/dl	0.9 (0.8 to 1.0)	0.8 (0.7 to 0.9)	1.2 (1.1 to 1.5)	0.0001

**Fig. 1:**
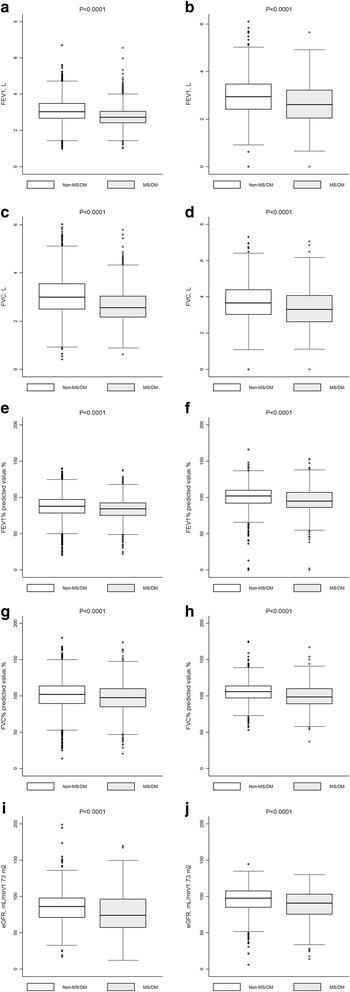
Distribution of forced expiratory volume in 1 s (FEV1), forced vital capacity (FVC), and estimated glomerular filtration rate (eGFR) by disease status. The figure shows the 25th, 50th (Median) and 75th percentile of the distribution of forced expiratory volume in 1 s (FEV1), forced vital capacity (FVC), and estimated glomerular filtration rate (eGFR) (vertical lines on each box). ‘Whiskers’ on each box indicate values at 1.5 times the interquartile range from the median and dots indicate the more extreme values, including the maximum and minimum of the distribution. MS, metabolic syndrome; DM, diabetes mellitus. **a** Distribution of FEV1 by disease status in Nanjing survey; (**b**) Distribution of FEV1 by disease status in Crossroads study; (**c**) Distribution of FVC by disease status in Nanjing survey; (**d**) Distribution of FVC by disease status in Crossroads study; (**e**) Distribution of FEV1% predicted value by disease status in Nanjing survey; (**f**) Distribution of FEV1% predicted value by disease status in Crossroads study; (**g**) Distribution of FVC% predicted value by disease status in Nanjing survey; (**h**) Distribution of FVC% predicted value by disease status in Crossroads study; (**i**) Distribution of eGFR by disease status in Nanjing survey; (**j**) Distribution of eGFR by disease status in Crossroads study

Figure 1 shows that the median (interquartile range) of FEV1, FVC, PFEV1, PFVC, and eGFR was 2.99 (2.50 to 3.56), 3.03 (2.67 to 3.49), 88 (78 to 97)%, 102 (90 to 114)% and 86.04 (71.01 to 97.92)% among Chinese participants and 2.94 (2.41 to 3.48), 3.66 (3.02 to 4.42), 102 (92 to 110)%, 106 (97 to 114)% and 88.43 (77.41 to 99.55)% among Australian participants without metabolic syndrome or type 2 diabetes, respectively.

Figure 1 also shows the median (interquartile range) of FEV1, FVC, PFEV1, PFVC, eGFR was 2.56 (2.16 to 3.04), 2.72 (2.41 to 3.05), 84 (75 to 93)%, 98 (85 to 110)% and 74.08 (56.99 to 96.45)% among Chinese participants and 2.61 (2.04 to 3.22), 3.30 (2.62 to 4.08), 95 (86 to 107)%, 99 (89 to 110)% and 83.98 (71.15 to 97.42)% among Australian participants with metabolic syndrome or type 2 diabetes, respectively.

**Fig. 2:**
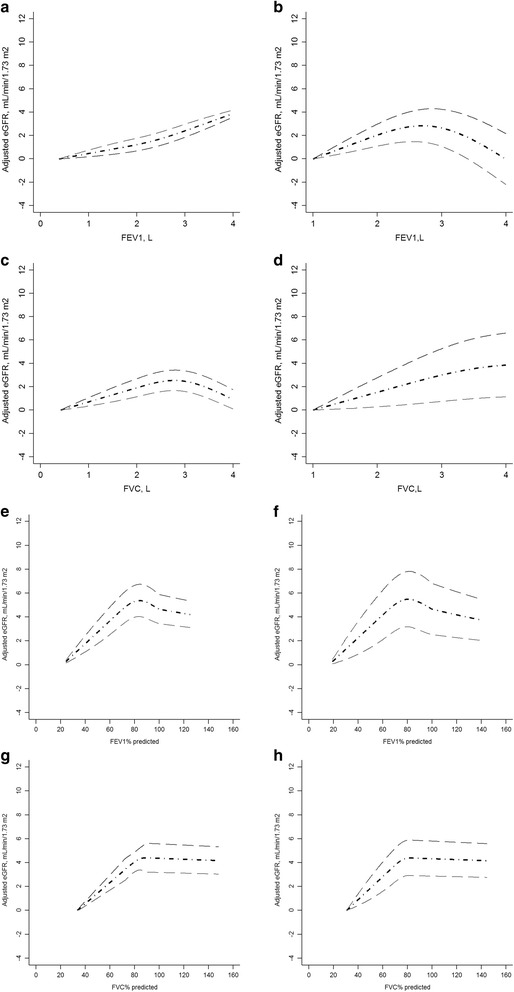
Dose-response relationship between adjusted estimated glomerular filtration rate (eGFR) and lung capacity measures. **a** Dose-response relationship between FEV1 and adjusted estimated glomerular filtration rate (eGFR) in Nanjing survey; (**b**) Dose-response relationship between FEV1 and adjusted estimated glomerular filtration rate (eGFR) in Crossroads study; (**c**) Dose-response relationship between FVC and adjusted estimated glomerular filtration rate (eGFR) in Nanjing survey; (**d**) Dose-response relationship between FVC and adjusted estimated glomerular filtration rate (eGFR) in Crossroads study. **e** Dose-response relationship between FEV1% predicted value and adjusted estimated glomerular filtration rate (eGFR) in Nanjing survey; (**f**) Dose-response relationship between FEV1% predicted value and adjusted estimated glomerular filtration rate (eGFR) in Crossroads study; (**g**) Dose-response relationship between FVC% predicted value and adjusted estimated glomerular filtration rate (eGFR) in Nanjing survey; (**h**) Dose-response relationship between FVC% predicted value and adjusted estimated glomerular filtration rate (eGFR) in Crossroads study; FEV1, forced expiratory volume in 1 s; FVC, forced vital capacity. People with minimum FEV1 or FVC was used as reference group. Age, gender, smoking status, waist circumference, systolic blood pressure, triglyceride, and high density lipoprotein cholesterol were adjusted

The relationship between lung capacity measures (FEV1, FVC, PFEV1, and PFVC) and renal function measure (eGFR) was presented in Fig. 2. eGFR linearly increased with FEV1 in the Chinese population and with FVC in the Australian population, respectively. The relationship between FEV1 and eGFR and between FVC and eGFR was non-linear among Australian participants and Chinese participants, respectively (both *P* < 0.0001 for linearity test). In both Chinese and Australian populations, the non-linear relationships were found between PFEV1 and eGFR and between PFVC and eGFR (all *P* < 0.0001 for linear test). The highest eGFR was found at 2.65 (95%CI: 2.42 to 2.88) L of FEV1 for Australian participants and 2.78 (95CI: 2.45 to 3.11) L of FVC for Chinese participants, respectively. The highest eGFR was found at 81.30 (80.21 to 82.40)% and 82.05 (80.67 to 81.36)% for PFEV1 in the Australian and Chinese participants, respectively. The highest eGFR was found at 82.56 (81.40 to 83.72)% and 84.32 (83.37 to 85.27)% for PFVC among Australian and Chinese participants, respectively.

Similar relationships were identified among people with normal metabolic status (Additional file 1: Figure S1). Among people with metabolic syndrome or type 2 diabetes, eGFR linearly increased with FEV1 and FVC in the Chinese population; in the Australian population, eGFR increased with FEV1 and peaked at 2.65 (2.42 to 2.88) L and declined thereafter; eGFR increased with FVC and plateaued at 3.05 (2.87 to 3.23) L. The PFEV1 linearly increased with eGFR both in Australian and Chinese population with normal metabolic status. The highest eGFR was found at 78.72 (77.81 to 79.63)% and 80.32 (79.18 to 81.46)% for PFVC among Australian and Chinese participants with normal metabolic status (Additional file 1: Figure S1), respectively. Among people with metabolic syndrome or type 2 diabetes, the highest eGFR was identified at 76.32 (75.38 to 77.26)% and 77.13 (76.17 to 78.09)% in Australian population and Chinese population respectively. The highest eGFR was identified at 82.56 (81.40 to 83.72)% only in Australian population. The association between PFVC and eGFR was non-linear with the sharp increase of eGFR at PFC more than 84.32 (83.37 to 85.27)% in Chinese population.

**Fig. 3:**
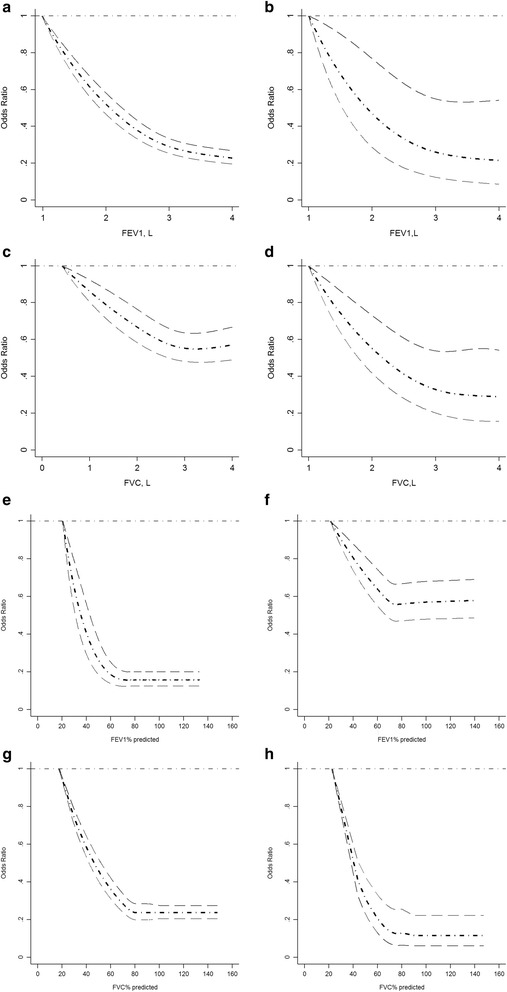
Dose-response relationship between adjusted odds ratio for reduced renal function and lung capacity measures. **a** Dose-response relationship between FEV1 and adjusted odds ratio for reduced renal function (eGFR < 60 mL/min/1.73 m^2^) in Nanjing survey; (**b**) Dose-response relationship between FEV1 and adjusted odds ratio for reduced renal function (eGFR < 60 mL/min/1.73 m^2^) in Crossroads study; (**c**) Dose-response relationship between FVC and adjusted odds ratio for reduced renal function (eGFR < 60 mL/min/1.73 m^2^) in Nanjing survey; (**d**) Dose-response relationship between FVC and adjusted odds ratio for reduced renal function (eGFR < 60 mL/min/1.73 m^2^) in Crossroads study. **e** Dose-response relationship between FEV1% predicted value and adjusted odds ratio for reduced renal function (eGFR < 60 mL/min/1.73 m^2^) in Nanjing survey; (**f**) Dose-response relationship between FEV1% predicted value and adjusted odds ratio for reduced renal function (eGFR < 60 mL/min/1.73 m^2^) in Crossroads study; (**g**) Dose-response relationship between FVC% predicted value and adjusted odds ratio for reduced renal function (eGFR < 60 mL/min/1.73 m^2^) in Nanjing survey; (**h**) Dose-response relationship between FVC% predicted value and adjusted odds ratio for reduced renal function (eGFR < 60 mL/min/1.73 m^2^) in Crossroads study; FEV1, forced expiratory volume in 1 s; FVC, forced vital capacity. People with minimum FEV1 or FVC was used as reference group. Age, gender, smoking status, waist circumference, systolic blood pressure, triglyceride, and high density lipoprotein cholesterol were adjusted

There was a non-linear relationship (Linearity test: both *P* < 0.0001) between FEV1 and adjusted odds ratios for reduced renal function (eGFR <60 ml/min/1.73m^2^), with clear evidence of a threshold estimated at 3.05 (95%CI: 2.82 to 3.28 L and 2.76 to 3.34 in both the Chinese and Australian populations respectively) (Fig. 3). Similar non-linear relationships (Linearity test: both *P* < 0.0001) were observed between FVC and adjusted odds ratio for reduced renal function and a threshold was identified at 3.05 (95%CI: 2.72 to 3.38 L and 2.68 to 3.41 L in both the Chinese and Australian populations respectively) (Fig. 3). A non-linear relationship was also found between PFEV and adjusted odds ratio for reduce renal function and between PFVC and adjusted odds ratio for reduced renal function with thresholds identified at 76.32 (75.38 to 77.26)% and 77.13 (76.17 to 78.09)% for PFEV1; 78.46 (77.55 to 79.37)% and 80.26 (79.00 to 81.52)% for PFVC, in Australian and Chinese population, respectively.

Among people with normal metabolic status, a non-linear relationship (Linearity test: *P* < 0.0001) between FEV1 and adjusted odds ratios for reduced renal function was found among Chinese participants with threshold at 3.05 (2.91 to 3.19) L. A linear relationship between FEV1 and adjusted odds ratios for reduced renal function was found among Australian participants. There was a non-linear relationship (Linearity test: both *P* < 0.0001) between FVC and adjusted odds ratios for reduced renal function (eGFR <60 ml/min/1.73m^2^), with clear evidence of a threshold estimated at 3.05 (95%CI: 2.87 to 3.23 L and 2.67 to 3.43 L in both the Chinese and Australian populations respectively) (Additional file 1: Figure S3). The odds ratios of reduced renal function decreased with the increase of PFEV1 in both Australian and Chinese populations. A non-linear relationship was observed between PFVC and adjusted odds ratios of reduced renal function both in Australian and Chinese population with thresholds at 78.72 (77.81 to 79.63)% and 80.32 (79.18 to 81.46)% for PFVC among Australian and Chinese participants with normal metabolic status (Additional file 1: Figure S3).

Among people with metabolic syndrome or type 2 diabetes, there was a non-linear relationship (Linearity test: both *P* < 0.0001) between FEV1 and adjusted odds ratios for reduced renal function, with clear evidence of a threshold estimated at 3.05 (95%CI: 2.91 to 3.19 L and 2.69 to 3.41 L in both the Chinese and Australian populations respectively) (Additional file 1: Figure S4). There was a non-linear relationship (Linearity test: both *P* < 0.0001) between FVC and adjusted odds ratios for reduced renal function, with clear evidence of a threshold estimated at 3.05 (95%CI: 2.87 to 3.23 L and 2.67 to 3.43 L in both the Chinese and Australian populations respectively) (Additional file 1: Figure S4). The odds ratios of reduced renal function decreased with the increase of PFEV1 in both populations with a clear threshold identified at 78.46 (77.55 to 79.37)% in the Chinese population. The non-linear relationship between PFVC and adjusted odds ratios of reduced renal function was similar in both populations with thresholds at 78.72 (77.81 to 79.63)% and 80.32 (79.18 to 81.46)% in Australian and Chinese populations, respectively.

The adjusted association between obstructive lung function and reduced renal function was presented in supplemental table-1. The increased adjusted odds ratio for reduced renal function was more likely to be found among people with obstructive lung function in both Chinese and Australian populations, especially for Chinese participants (all *P* < 0.0001). Interaction between metabolic disorder and obstructive lung function on the increased adjusted odds ratios for reduced renal function was both identified in Chinese and Australian population, especially for Chinese participants (all *P* < 0.0001).

## Discussion

We focused our investigation on the shape of the relationship between pulmonary function measurements and risk of reduced renal function in a Chinese sample and repeated the analyses in an Australian population, assessing the evidence for non-linearity and, in particular, on the existence of a threshold. In our analyses, we found consistent evidence that the associations between lung capacity measures and risk of reduced renal function are non-linear. Analyses demonstrated a threshold for reduced renal function of 3.05 L both for FEV1 and FVC in both the Chinese and Australian populations. It was also identified that obstructive lung function, especially interacted with metabolic disorders was associated with increased risk of reduced renal function.

Few studies have addressed the association between FEV1 and FVC as lung function markers and eGFR in representative populations. The National Health and Nutrition Examination Survey 2007–2012 investigated 7610 participants aged 40–79 years and revealed that impaired lung function is common in those with and without chronic kidney disease, and eGFR <60 ml/min/1.73 m2 was associated with higher odds of obstructive lung function [3]. In another small Japanese sample, it was also found that decreased eGFR is associated with decreased pulmonary function markers of pulmonary diffusion capacity [21]. In another cohort study incorporating vascular surgery patients, chronic obstructive pulmonary disease (COPD) was found to be associated with restricted renal function and chronic kidney disease [22]. Consistent with these findings, we have found an association between restricted pulmonary function and impaired renal function in both a Chinese population and reproduced this finding in an Australian population.

However, the underlying association between lung capacity measurements and risk of impaired renal function have been assumed to be linear. Few studies have investigated the possible exposure-response association between pulmonary function measurements and renal function measurements, nor the exposure-response association between lung function and risk of impaired renal function. The potential mechanism might be that the systemic inflammation might trigger the restricted lung function and the progression of atherosclerosis which leading to the reduced renal function [23, 24]. The non-linear relationship between lung capacity and renal function might reflect the development process of systemic inflammation, as irreversible consequence like COPD or chronic kidney disease could rapidly progressed once atherosclerosis developed to a certain level [25].

Our study is the first study that has investigated both the exposure-response association between pulmonary capacity and renal function and explored the exposure-response association between pulmonary function and risk of impaired function within two independent samples from China and Australia. Previous findings were extended by our study, as there is a non-linear association between pulmonary capacities (especially FEV1) and impaired renal function: The risk of impaired renal function was increased below 3.05 L FEV1 and FVC. And this finding was remarkably consistent over the samples from China and Australia.

We wonder if the information from this study indicates that patients with obstructive lung disease below the threshold of 3.05 L should be screened for impaired renal function. Using an assumption of linearity in the exposure-response association, there are no potential thresholds of pulmonary function measurements, and therefore, it was previously difficult to propose a threshold for heightened awareness of the risk of significant renal function impairment. However in this refined exploration of the exposure-response association and thresholds, a threshold with relative precision has been generated.

Comparisons of independent samples between countries provides an opportunity to investigate the similarities and differences in the association between pulmonary function and impaired renal function. Both cross-sectional samples incorporate variation by methodology, ethnicity, and medical systems, potentially leading to variation in the clinical measurements and prevalence of disease. Therefore, instead of merging these datasets, we explored the associations in one independent sample and replicated the analyses in the other independent sample.

There might also be underlying differences in the distribution of pulmonary function and renal function between two countries and in the delivery and effectiveness of healthcare, contributing to observed difference in association between pulmonary function and renal function measurements, for example, in the Australian population, the highest eGFR was identified among those with FEV1 of 2.65 L however it was observed at 2.78 L in the Chinese population. The differences observed in this study were expected and might also be partially explained by the ethnic differences and the smaller Australian sample size. The ethnic differences could reflect distinguishing genetic backgrounds and anthropometry. However it was feasible to compare associations between countries by applying a rigorous approach to data analysis.

The approach we have used included a comparison of participants’ characteristics, levels of lung and renal function measurements, the association between pulmonary function measurements and renal function measurements, and the association between pulmonary function measurements and impaired renal function. This indicates that the two samples in the two countries were comparable and this was supported by the consistency in the association between pulmonary function measurements and renal function measurements between the two samples.

One possible explanation for the association between impaired pulmonary function and reduced renal function observed in this study may be underlying systemic inflammation. A systemic inflammatory response and accumulation of pro-inflammatory cytokines have been postulated to modulate progression of both glomerular and tubule-interstitial scarring, leading to reduced renal function [26]. Pro-inflammatory cytokines may also influence the rate of muscle and protein breakdown, leading to the development of impaired pulmonary lung function [27].

The utilisation of cross-sectional data is the main limitation in our study, whereby pulmonary function and diseases were measured at the same time. Therefore it would be difficult to deduce the causal association between pulmonary function and impaired kidney function, especially if the progression of disease had an impact on the pulmonary function. Prospective study would be the next step to investigate these associations. Another limitation is that the data used in this joint study were not collected within the same survey: the data from Chinese and Australian samples are c0mparable though. The temporary difference in Chinese and Australian data collection might also have some influence on the research samples.

## Conclusion

In summary, our results suggest a non-linear relationship between pulmonary function measurements and risk of impaired renal function exists in both Chinese and Australian populations. The thresholds of FEV1 and FVC for the lowest risk of impaired renal function was above 3.05 L (95%CI: 2.82 to 3.28 L and 2.76 to 3.34 L for FEV1 in Chinese and Australian population respectively; 2.72 to 3.38 L and 2.68 to 3.41 L for FVC in Chinese and Australian population respectively). FEV1 or FVC below 3.05 L, with PFEV1 below 76–77% or with PFVC below 79–80%, respectively, was associated with a high risk of impaired renal function. Obstructive lung function was associated with a high risk of impaired renal function. Interaction between obstructive lung function and metabolic disorders was associated with the highest risk of impaired renal function.

## Additional file

Additional file 1: Figure S1. Dose-response relationship between adjusted estimated glomerular filtration rate (eGFR) and lung capacity measures among people with normal fasting glucose. **Figure S2.** Dose-response relationship between adjusted estimated glomerular filtration rate (eGFR) and lung capacity measures among people with metabolic syndrome or type 2 diabetes. **Figure S3.** Dose-response relationship between adjusted odds ratios for reduced renal function and lung capacity measures among people with normal fasting glucose. **Figure S4.** Dose-response relationship between adjusted odds ratios for reduced renal function and lung capacity measures among people with metabolic syndrome or type 2 diabetes. (DOCX 125040 kb)

## Abbreviations

AIC: Akaike information criterion
CKD-EPI: Chronic Kidney Disease Epidemiology Collaboration
COPD: chronic obstructive pulmonary disease
eGFR: estimated glomerular filtration rate
FEV1: Forced expiratory volume in one second
FVC: Forced vital capacity
IDF: International Diabetes Federation
MS: Metabolic syndrome
NHANES: National Health and Nutrition Examination Survey
P5: 5th percentile
P95: 95th Percentile
