# Supplementary material for: Association between pulmonary function and renal function: findings from China and Australia
Source: BMC Nephrol. 2017 May 1;18:143. doi: 10.1186/s12882-017-0565-y (PMC5412033; doi:10.1186/s12882-017-0565-y)
Supplement: Additional file 1: Figure S1. — Dose-response relationship between adjusted estimated glomerular filtration rate (eGFR) and lung capacity measures among people with normal fasting glucose. Figure S2. Dose-response relationship between adjusted estimated glomerular filtration rate (eGFR) and lung capacity measures among people with metabolic syndrome or type 2 diabetes. Figure S3. Dose-response relationship between adjusted odds ratios for reduced renal function and lung capacity measures among people with normal fasting glucose. Figure S4. Dose-response relationship between adjusted odds ratios for reduced renal function and lung capacity measures among people with metabolic syndrome or type 2 diabetes. (DOCX 125040 kb) [file 12882_2017_565_MOESM1_ESM.docx]

**Online supplemental**

Supplemental Figure-1. Dose-response relationship between adjusted estimated glomerular filtration rate (eGFR) and lung capacity measures among people with normal fasting glucose

*(a) Dose-response relationship between FEV1 and adjusted estimated glomerular filtration rate (eGFR) in Nanjing survey;*

*(b) Dose-response relationship between FEV1 and adjusted estimated glomerular filtration rate (eGFR) in*

*Crossroads study;*

*(c) Dose-response relationship between FVC and adjusted estimated glomerular filtration rate (eGFR) in Nanjing survey;*

*(d) Dose-response relationship between FVC and adjusted estimated glomerular filtration rate (eGFR) in*

*Crossroads study;*

*(e) Dose-response relationship between FEV1% predicted value and adjusted estimated glomerular filtration rate (eGFR) in Nanjing survey;*

*(f) Dose-response relationship between FEV1% predicted value and adjusted estimated glomerular filtration rate (eGFR) in Crossroads study;*

*(g) Dose-response relationship between FVC% predicted value and adjusted estimated glomerular filtration rate (eGFR) in Nanjing survey;*

*(h) Dose-response relationship between FVC% predicted value and adjusted estimated glomerular filtration rate (eGFR) in Crossroads study;*

*FEV1, forced expiratory volume in 1s; FVC, forced vital capacity. People with minimum FEV1 or FVC was used as reference group. Age, gender, smoking status, waist circumference, systolic blood pressure, triglyceride, and high density lipoprotein cholesterol were adjusted.*

Supplemental Figure-2. Dose-response relationship between adjusted estimated glomerular filtration rate (eGFR) and lung capacity measures among people with metabolic syndrome or type 2 diabetes

*(a) Dose-response relationship between FEV1 and adjusted estimated glomerular filtration rate (eGFR) in Nanjing survey;*

*(b) Dose-response relationship between FEV1 and adjusted estimated glomerular filtration rate (eGFR) in*

*Crossroads study;*

*(c) Dose-response relationship between FVC and adjusted estimated glomerular filtration rate (eGFR) in Nanjing survey;*

*(d) Dose-response relationship between FVC and adjusted estimated glomerular filtration rate (eGFR) in*

*Crossroads study.*

*(e) Dose-response relationship between FEV1% predicted value and adjusted estimated glomerular filtration rate (eGFR) in Nanjing survey;*

*(f) Dose-response relationship between FEV1% predicted value and adjusted estimated glomerular filtration rate (eGFR) in Crossroads study;*

*(g) Dose-response relationship between FVC% predicted value and adjusted estimated glomerular filtration rate (eGFR) in Nanjing survey;*

*(h) Dose-response relationship between FVC% predicted value and adjusted estimated glomerular filtration rate (eGFR) in Crossroads study;*

*FEV1, forced expiratory volume in 1s; FVC, forced vital capacity. People with minimum FEV1 or FVC was used as reference group. Age, gender, smoking status, waist circumference, systolic blood pressure, triglyceride, and high density lipoprotein cholesterol were adjusted.*

Supplemental Figure-3. Dose-response relationship between adjusted odds ratios for reduced renal function and lung capacity measures among people with normal fasting glucose

*(a) Dose-response relationship between FEV1 and adjusted odds ratio for reduced renal function (eGFR<60 mL/min/1.73 m^2^) in Nanjing survey;*

*(b) Dose-response relationship between FEV1 and adjusted odds ratio for reduced renal function (eGFR<60 mL/min/1.73 m^2^) in Crossroads study;*

*(c) Dose-response relationship between FVC and adjusted odds ratio for reduced renal function (eGFR<60 mL/min/1.73 m^2^) in Nanjing survey;*

*(d) Dose-response relationship between FVC and adjusted odds ratio for reduced renal function (eGFR<60 mL/min/1.73 m^2^) in Crossroads study.*

*(e) Dose-response relationship between FEV1% predicted value and adjusted odds ratio for reduced renal function (eGFR<60 mL/min/1.73 m^2^) in Nanjing survey;*

*(f) Dose-response relationship between FEV1% predicted value and adjusted odds ratio for reduced renal function (eGFR<60 mL/min/1.73 m^2^) in Crossroads study;*

*(g) Dose-response relationship between FVC% predicted value and adjusted odds ratio for reduced renal function (eGFR<60 mL/min/1.73 m^2^) in Nanjing survey;*

*(h) Dose-response relationship between FVC% predicted value and adjusted odds ratio for reduced renal function (eGFR<60 mL/min/1.73 m^2^) in Crossroads study.*

*FEV1, forced expiratory volume in 1s; FVC, forced vital capacity. People with minimum FEV1 or FVC was used as reference group. Age, gender, smoking status, waist circumference, systolic blood pressure, triglyceride, and high density lipoprotein cholesterol were adjusted.*

Supplemental Figure-4. Dose-response relationship between adjusted odds ratios for reduced renal function and lung capacity measures among people with metabolic syndrome or type 2 diabetes

*(a) Dose-response relationship between FEV1 and adjusted odds ratio for reduced renal function (eGFR<60 mL/min/1.73 m^2^) in Nanjing survey;*

*(b) Dose-response relationship between FEV1 and adjusted odds ratio for reduced renal function (eGFR<60 mL/min/1.73 m^2^) in Crossroads study;*

*(c) Dose-response relationship between FVC and adjusted odds ratio for reduced renal function (eGFR<60 mL/min/1.73 m^2^) in Nanjing survey;*

*(d) Dose-response relationship between FVC and adjusted odds ratio for reduced renal function (eGFR<60 mL/min/1.73 m^2^) in Crossroads study.*

*(e) Dose-response relationship between FEV1% predicted value and adjusted odds ratio for reduced renal function (eGFR<60 mL/min/1.73 m^2^) in Nanjing survey;*

*(f) Dose-response relationship between FEV1% predicted value and adjusted odds ratio for reduced renal function (eGFR<60 mL/min/1.73 m^2^) in Crossroads study;*

*(g) Dose-response relationship between FVC% predicted value and adjusted odds ratio for reduced renal function (eGFR<60 mL/min/1.73 m^2^) in Nanjing survey;*

*(h) Dose-response relationship between FVC% predicted value and adjusted odds ratio for reduced renal function (eGFR<60 mL/min/1.73 m^2^) in Crossroads study.*

*FEV1, forced expiratory volume in 1s; FVC, forced vital capacity. People with minimum FEV1 or FVC was used as reference group. Age, gender, smoking status, waist circumference, systolic blood pressure, triglyceride, and high density lipoprotein cholesterol were adjusted.*
